# American Dental Patents

**Published:** 1855-01

**Authors:** Geo. W. Kendall


### ARTICLE IV.

### *American Dental Patents.* By GEO. W. KENDALL.

Continued from page 421, vol. 3.

MESSRS. EDITORS—So much has been said upon the patent question within the last two years, and as the profession generally appears to be opposed to patenting dental improvements, I will not trespass upon your pages to extend the argument, but give a *resume* of the dental patents which have been issued since the appearance of my last paper on this subject.

Of these, the most important is that granted to A. J. Watts, of Utica, N. Y., for a mode of preparing sponge gold for filling teeth. The claim and specification has already appeared in the Journal, and need not be here repeated.

The claim covers the process of amalgamating pure gold with mercury, dissolving out the mercury in nitric acid, washing and heating the residuum.

Almost immediately upon the issuing of the patent, H. R. White & Co., of Utica, and Taft & Watts, of Xenia, Ohio, presented prepared gold to the profession, apparently made in the same manner as that of the patentee. That of Watts and White is a light, bulky mass of a dark brown color assuming the metallic lustre upon compression or friction.

Taft & Watts', however, is more highly crystalline in its character, or rather it is composed of larger crystals, and approaches nearer the familiar color of pure gold, (and is therefore called by them CRYSTAL GOLD.) It has been intimated that it is prepared by following the general process of the patent, and that its peculiar character depends upon some manipulations, not generally understood.

(These gentlemen were urged to explain the process at a meeting of the Mississippi Valley Association, but declined to do so, promising at some future time to give it publicity. That time has not yet arrived, unless perchance it has been developed at the present session of the Ohio Dental College, in which one of the gentlemen is now a professor.)

It has a peculiar harshness to the touch and crepitates slightly when a cake of it is bent or broken; characteristics communicated, most probably, by some alloy of the baser metals found in those specimens subjected to chemical analysis.

Every variety of sponge gold that I have seen will absorb moisture from the air into its pores, and is, therefore, much improved by heating slightly before using.

It is not to be supposed that the sponge gold will entirely supersede gold foil, but for many cavities I think it will come into very general use; but it has been discussed at such length elsewhere that I will leave it.

*Lewis F. Sheppard*, Alhambra, Illinois.—This is a patent for which an application was pending at the time of writing my previous article, and to which allusion was there made. The claim is for extending a suitable metallic plate over the masticating portion of artificial teeth to protect them from injury by use, &c.

*Melvin Jenks*, Wayland, N. Y., Dec. 13, 1853.—For an improvement in turnkeys.

*A. Merrett Asay*, Philadelphia, February, 1854.—For improved dental chair. The claim is for "moving the seat vertically by means of screws, wheels, rack and arms as set forth."

*F. Davison*, January 17th, 1854.—Saliva pump. The claim is for drawing the saliva from the mouth and keeping it dry, during the operation of filling teeth, by means of an instrument constructed with a hollow mouth-piece, which connects with a tube, suction and force pump.

*Henry L. Crider & D. Williams*, Lancaster, O., Oct. 18, 1853.—The claim is for soldering single gum teeth to a narrow plate, fitted to the ordinary plate, along the alveolar ridge, and fastening the two together by riveting or soft solder, so as to prevent the springing of the plate in soldering.

Dr. Harvey Sellers exhibited a set of teeth made precisely in that way in this city in 1851, and spoke of it as an excellent plan, and one frequently adopted by him. This one single set of teeth made two years prior to the issue of the patent, is sufficient to invalidate the patent, but as the patentees can show earnest and strong recommendations from D. D. S's, and M. V. A. D. S's, and the price of an office-right is trifling, (varying from $5 to $50,) quite a number of dentists have been induced to purchase rights, and pay just so much *black mail.*

*Mahlon Loomis*, Cambridgeport, Mass., May 2d, 1854.—"For an improvement in the manufacture of artificial teeth."

The improvement consists in making complete dentures of porcelain, dispensing entirely with metal plates. The specification occupies seven foolscap pages, and is exceedingly verbose, indefinite and unintelligible. I will, however, give a summary.

*Thin* plates of silver or other proper metal are got up in the usual way and fitted to the mouth perfectly. Upon these, wax is arranged, to indicate the desired length and contour of the denture, or dentures, and a plaster cast taken inside of this plate. In a very prolix way he says that porcelain paste contracts in baking, and different materials and proportions vary the contraction; he estimates the contraction as a general thing at one-sixteenth of the whole bulk, but directs us to ascertain the amount of shrinkage of the body which is to be employed, by measurement and experiment.

The plaster cast is now to be enlarged in the same proportion that the body shrinks. I will quote his method of enlarging the cast.

"This (the amount of shrinkage) being ascertained, the aforesaid plaster cast *may be scooped out sufficiently to allow for shrinkage,* and it may be extended back the proportional distance from the mark left by the rear edge of the plate to allow for longitudinal shrinkage."

"The wax is now removed from the metal plate, and placed on the plaster cast, the deficiency at its two extremities being supplied if necessary, its height and breadth corrected, if requisite, by the addition of wax."

"Over the wax model and the remaining surface of the face of the cast, plaster is poured; when hardened, this plaster is cut away so as to form a space between it and the face of the cast, which will admit of the porcelain mixture, running back far enough to force that portion of the upper set which is usually made of metallic plate."

☞ "The space thus made between the masses of plaster, constitutes the matrix for forming the upper portions of the set of teeth."

The matrix for forming the lower set is made in a similar manner.

"The enlargement may be done by sawing the cast apart crosswise, and next gluing the parts firmly together with a piece of wood between them, which shall correspond in thickness with the amount of shrinkage of the porcelain material in a longitudinal direction. Next, the whole may be cut apart at right angles with the first separation, and again glued together, with a similar strip of wood, to allow for *transverse* shrinkage.

The surface of the plaster mould is now coated with bayberry tallow, and the porcelain paste packed in and carved and shaped as may be desired. "The plaster mould is now removed by heating the whole mass, and the '*cast*' (formed in the matrix) is placed upon the tile for baking in the usual way. After the two portions have been taken from the furnace the *last* time, they may be ground on their inside surfaces to make them fit a plaster cast of the mouth." Next follows a description of the application of the plan to partial sets of peculiar difficulty, and here is the claim: "I do not claim the process as above set forth in making sets of artificial teeth; I do not claim the spreading of a gum enamel over one side of a metallic roof-plate upon which the teeth are fastened; nor the extension of the porcelain gum some way, and not entirely upon the roof; but, what I claim is the improved manufacture of whole or half sets of porcelain or mineral teeth substantially as described."

The claim is certainly a most singular one, and I cannot understand precisely what it is that the patent is granted for, as the method is expressly disclaimed. I suppose that it is for the result of the process, viz. a complete denture of porcelain; and we have precedents fully as ridiculous in our list of dental patents. The very first attempt at making porcelain teeth, of which we have any account, was that of M. Duchateau, who had a set of ivory teeth from which he suffered a good deal of pain, and he conceived the idea of removing them and substituting one of porcelain, and for this purpose applied to M. de Chemaut, a Parisian dentist. After many attempts, he obtained a piece of grayish white, very little warped and capable of being used.

The Academy of Medicine, to which he communicated his discovery, voted Duchateau their thanks and granted him the honor of a seat.

M. de Chemaut improved Duchateau's method, and procured a patent for porcelain teeth without plates.

Audibrand, Desforges, Maury, Lefoulon, and nearly every other French writer on artificial teeth, speak of such operations.

The work of the latter was translated by Professor Bond and published in the Am. Lib. Dental Science in 1844, and on page 280, under the head of "complete dentures of porcelain paste," will be found a much more complete description than that given by Mr. Loomis in his patent specifications. He then proposes to enlarge the cast by many successive mouldings in plaster, the swelling of which causes each mould to be larger than the preceding. This enlarges the cast not merely in two lines, but proportionally in every part and point. He, however, prefers to enlarge the cast by a process known in statuary, by which busts or statues are increased or diminished.

I confess that I am totally at a loss to understand how Mr. Loomis can enlarge the upper cast, by scooping it out; or how cutting away a portion inside of the alveolar ridge can overcome the contraction outside of the ridge.

The division of the lower jaw into sections and separating, will nearly counteract the contraction in cases when absorption is very great and the jaw very flat. I know of a case which was made in this way, ten years ago, and answered an excellent purpose. Those who have had any experience in block work, will be quite sceptical about the possibility of carving and handling a complete denture of porcelain paste without breaking, unless it be too bulky to be worn in the mouth. They know that the warping of an ordinary block, of six front teeth, is not an unfrequent occurrence, and will be quite incredulous, when they hear that *scooping out* a cast will enable them to carve and burn a full arch, which shall not be injured by warping or contraction.

Although Lefoulon increased his cast in every proportion, and used a peculiar body, and says he has made great use of this kind of operation with satisfaction to his patients, he remarks, with amusing naivetté, "it is prudent to make at least six pieces at a time, for of the six there is no security that the fire will leave one without crack or torsion!"

An insuperable objection to the general adoption of this plan, is the necessity of carving, for very few dentists are possessed of the requisite natural talent, or have the necessary practice to produce blocks of the ordinary style suitable for the mouth.

And even in the best hands, the work must be heavy and clumsy, easily broken, and difficult to repair, and *practical work cannot be made by following the specifications.*

Although very proud of the inventions and improvements of our Yankees, and though always willing to huzza and throw up my hat upon the announcement of any real improvement of Yankee origin, I have not the slightest disposition to join in the jubilant welcome this one has met, but would rather class it with the horn gun-flints and wooden nutmegs, and things of that ilk, which have conferred such an unenviable notoriety upon Yankee doodledom.

*Lorenzo Simmonds*, Boston, Sept. 12th, 1854.—The claim is "for attaching to an artificial palate, or to any plate to be secured in the mouth, an air-chamber constructed with a flexible elastic diaphragm for more effectually exhausting the air from between the plate and roof of the mouth."

I have been unable to procure the specifications of this patent as yet from the office.

It will doubtless be remembered by many, that soon after the publication of Dr. Hunter's description of his mode of constructing teeth with continuous gums, Dr. A. Hill described, in the Dental Recorder, (Oct., 1851,) a method of setting artificial teeth by inserting them in a base of "Hill's stopping" moulded upon the cast, and recently has described the method at greater length.

In 1848, *three years previous*, a patent was granted in England, to Edward Truman, dentist, of London, for a method of mounting artificial teeth, in which, the teeth were first mounted on a kind of skeleton frame work, which was then enveloped in gutta-percha and moulded upon a cast of the mouth. This was then gilded, and hence the name *auroplastic.* A suit for infringement of this patent, was recently heard in one of the English courts, and upon the defendant showing that on one occasion prior to the issue of the patent, a dentist had repaired a set of teeth, (rendered useless by great absorption subsequent to their insertion,) by placing gutta-percha between the plate and gum and up against the artificial teeth, replaces the parts lost by absorption, the case was dismissed and the patent declared invalid. The reader may judge how difficult it would be to sustain certain American patents.

But what I wish more particularly to call to your attention, is the intimation given by Dr. Hill in one of his articles, that the patent for "Hill's stopping," covers the application of that compound to "auroplastic bases." He says that the compound for the *base* is the "stopping," colored with red oxyd of iron, rose red, rouge, calcined asbestos or other similar substances. After telling us that these were his coloring materials, it was quite gratuitous on his part, to tell us further, that the material would not retain its color in the mouth, for the merest tyro in dental chemistry knows that oxyd of iron will not resist the action of the buccal fluids, and that rose red or calcined asbestos will not communicate a gum color to gutta-percha. But this is a fair sample of the practical working of the patent system in our profession.

Allowing that Dr. Hill's rights are all that he seems to assume, (and he can easily obtain a patent for the invention,) that the method is useful and practicable, yet the want of a proper gum color will prevent its use.

The patentee (?) cannot make a proper article, and any one who might be able to give the profession a mixture of gutta-percha, with substances as would give it the requisite hardness and color, would be deterred from doing so, by a fear of a prosecution for infringment of patent, with all its harrassing vexations and heavy expenses.

It is just like the dog in the manger, who could not eat the hay, nor would he let the ox eat it.

Perhaps this is all consistent with "high professional integrity and the soul of honor," as understood in "Yankee doodledom," but out in these primitive wilds, I can assure you it would be very differently denominated.

But the patent was for a compound for filling teeth, and that only, and it is no more an infringment of that patent, to make, use and sell a composition identical with "Hill's stopping" *for mounting artificial teeth*, than to use it in the manufacture of a baby's rattle box.

The inference made by me in a former article, to the action of the Mississippi Valley Association, and of the Ohio College, in relation to the patent question, has been construed into an opposition on my part to all dental colleges and societies, by certain parties. To all of that canting crew I can say, that I believe that properly conducted societies, are the most efficient means of fostering, developing and perfecting new and useful improvements, of elevating the professional standard, and of suppressing dental quackery; that a well conducted dental college affords great and unequaled advantages for the acquisition of a knowledge of the theories and principles necessary in a professional education. But I do not, therefore, believe that a society is at all conducive to the welfare of the profession, when its influence can be secured to aid in a bold fraud and unscrupulous outrage upon the rights of an individual, and of the profession at large; or that the "stock of knowledge" will be much increased by the reiteration of old and well established theories or principles of practice.

Nor do I believe, that an institution is calculated to elevate the profession much, when it confers its highest honors (?) alike upon the talented, well educated gentleman, and upon the ignorant and self-conceited ass, provided that each complies with its pecuniary requisitions; nor that there can be any more certain way devised to destroy all confidence in dental colleges than the multiplication or continuation of those in which money, and not merit, in professional knowledge is the essential prerequisite for graduation; where an unfortunate ambition prevails to boast of a large class and a long list of graduates; where every candidate receives a diploma, and the whole history of which cannot point out a single rejected candidate!

In a former article reference was made to a meeting of the Mississippi Valley Association as "the last," the article, however, did not appear until another meeting had been held, and as you had stricken out the date from the manuscript, I was made to speak of the meeting of '53 instead of '52, as was my intention. I also fell into the error of regarding a lecturer as a professor, and speaking of him as such.

Under the pretext of correcting these "gross and unjust errors," appeared a three page editorial, charging me with prevarication, wilful and deliberate falsification, and many other sins of omission and commission, and to cap the climax of the gentleman's indignation, it was asserted that I had actually accepted a *gift* of two tickets admitting me to a course of lectures in the Dental College of Ohio!

The whole article was as funny as one of Mrs. Partington's effusions, and the editor very much like that venerable dame, who says, that whenever she opens her mouth, she puts her foot in it.

The somewhat celebrated case of Allen *vs.* Hunter, for an infringemet of Allen's patent for continuous gum work, was to have been tried at the October session of the District Court. Dr. Hunter appeared and asked that the case should be heard; Dr. Allen appeared and asked for a continuance, as he was not ready for trial, citing divers technicalities and quibbles therefor; a continuance was granted, and the case will again come up in April, so the patentee has six months extension of the time in which he can sell *rights* to use a mode, which after that time will be the common property of the whole profession unencumbered and untrammeled.

I have heard that wicked and bad men or women sometimes trump up serious but fictitious charges against innocent parties, and make a somewhat plausible showing for the purpose of coercing them into the payment of money down, rather than stand the greater expense of a lawsuit. Money so obtained is termed BLACK MAIL, will you be so kind as to point out the difference between it, and the money obtained by the holder of a patent which is soon to be declared null and void when it comes to trial?

CINCINNATI, *Nov.* 1*st*, 1854.

---

